# Aging Reduces the Expression of Lung CINC and MCP-1 mRNA in a *P. aeruginosa* Rat Model of Infection

**DOI:** 10.1007/s10753-014-9813-5

**Published:** 2014-01-25

**Authors:** Jie Wen, Cheng-Mei Li, Li Gu, Shao-jun Yin, Wei Li, Rong Yang

**Affiliations:** 1 Department of Respiratory Medicine, Traditional Chinese Medical of Xinjiang Uygur Autonomous Region, Xinjiang, 830000 China; 2 Department of Paediatrics, Tenth People’s Hospital, Tongji University, Shanghai, China; 3 Department of Respiratory Medicine Shanghai 6th People’s East Hospital, Jiaotong University School of Medicine, Pudong Nanhui New City, West Three Lake Road No. 222, Shanghai, China; 4 Department of Geriatric Medicine, Tenth People’s Hospital, Tongji University, Shanghai, China

**Keywords:** chemokines, elderly, lung infection, CINC mRNA, MCP-1 mRNA

## Abstract

We investigated dynamic changes of inflammatory cell infiltration and expression of cytokine-induced neutrophil chemoattractant (CINC) and monocyte chemoattractant protein-1 (MCP-1) mRNA in aged rats with *Pseudomonas aeruginosa* pulmonary infection. Disease manifestation and lung tissue pathology (lesion dispersion, inflammatory reactions, tissue edema and bleeding) were more severe in aged rats than young rats. At various time points, lung tissue polymorphonuclear neutrophil and mononuclear macrophage numbers were lower in the aged group than the young group (*P* < 0.05), and at 24 h there was no difference in mononuclear macrophage numbers. After inoculation with *P. aeruginosa*, CINC and MCP-1 mRNA expression increased in both groups, but the peak lagged in old rats compared with young. Thus, aging can reduce the expression of CINC and MCP-1 mRNA in lung tissues, and reduce the infiltration of neutrophils and monocyte–macrophages induced by CINC and MCP-1. This might lead to increased risk of pneumonia in elderly patients.

## INTRODUCTION

Leukocyte migration from the blood into tissues in the context of inflammation is a complex phenomenon representing the integration of chemoattraction. Chemokines are a family of polypeptides with four conserved cysteine residues that form two disulfide bonds. The family can thus be subdivided into two large groups, depending on whether there is an intervening amino acid between the first two cysteines yield in the CXC and CC subgroups, respectively [1–3].

Generally, members of the CXC chemokines are chemotactic for neutrophils and CC chemokines are chemotactic for monocytes and a small subset of lymphocytes. Cytokine-induced neutrophil chemoattractants (CINC) are particular members of the CXC family that are active as neutrophil chemoattractants *in vitro* and *in vivo*. Monocyte chemoattractant protein-1 (MCP-1) is the major lymphocyte chemoattractant secreted by mitogen-stimulated peripheral blood mononuclear cells and acts as a potent T-lymphocyte and monocyte chemoattractant.

In recent years, with increasing socio-demographic aging, the number of aging infected patients has quickly increased, becoming a global problem [4]. The lung is frequently a site of infection in elderly patients. Studies have shown that altered cytokine systems due to aging can affect the pathophysiology of elderly pneumonia, but whether cytokines in the lung are also affected by aging is not clear. In this study, we used bacteriology, pathology and molecular biology to investigate the dynamic changes of inflammatory cell infiltration and the expression of CINC mRNA and MCP-1 mRNA in aged rats with *Pseudomonas aeruginosa* pulmonary infection. This might help us understand the possible mechanisms of cell penetration kinetics of lung inflammation, and help establish a basis for finding new treatment targets for aging pneumonia.

## MATERIALS AND METHODS

### Animals

Sixty-eight male Sprague–Dawley rats were divided into two groups: old group and young group. Rats in the old group (*n* = 34), which were purchased from Beijing Vital River Laboratories, were 20–22 months old and weighed 700–900 g. The young group (*n* = 34) consisted of rats that were purchased from the Experimental Animal Center of Fudan University, were 5–6 months old, and weighed 460–580 g. All animals had unified numbering and were housed under specific pathogen-free conditions. Animals were maintained and used in accordance with the animal use regulations A-75-10-01.

### *Pseudomonas aeruginosa*

*P. aeruginosa* standard strain (ATCC27853) was purchased from Zhongshan Hospital, Fudan Experimental Center of Clinical Microbiology, and was grown 35 °C MH agar plate for 24 h, then Maxwell tube and spectrophotometer colorimetry (wavelength 550 nm, absorbance 0.5) was performed using a two Maxwell unit concentration (6 × 10^8^ cfu/ml) with sterile distilled water (most *P. aeruginosa*-induced pneumonia models used this concentration of bacteria [5, 6]).

### Drugs and Reagents

A solution of 0.9 % sodium chloride (Shanghai Changzheng Fumin Jinshan Pharmaceutical Co., Ltd., China) and 3 % pentobarbital sodium solution (Shanghai Xitang Biotechnology Co., Ltd., China) were used. Other reagents used were as follows: Anerdian (Shanghai Li Kang Hi-Tech Co., Ltd. China), 10 % buffered formalin (Shanghai Tenth People's Hospital Central Laboratory, China), MH agar plates (Shanghai Reagent Supply Research Center products, China), Xylene (Shanghai Reagent Supply Research Center products, China), diethylpyrocarbonate (DEPC; Shanghai Biological Engineering Technology Company, China), DEPC-treated water (Shanghai SBS Genetech, Inc., China), Trizol RNA extraction kit (Invitrogen Corporation, USA), SYBRGreen PCR kit (Shanghai Dawei Biotechnology Company, China), PCR Primers (Shanghai Runna Biotechnology Co., Ltd. China), chloroform (HPLC grade; Shanghai Reagent Factory, China), isopropanol (HPLC grade; Chemical Plant in Suzhou Zhenya, China), and ethanol (AR grade, Shanghai Zhenxing Chemical Plant, China).

### Disease Modeling

All experimental animals were divided into an aged group (experimental group) and young group (control group). Each group consisted of 24 rats and was divided into six subgroups at random: 0 (before inoculation), 2, 6, 9, 12 and 24 h. Every sub-group contained four rats. Then the animal model was established by tracheal inoculation with *P. aeruginosa* and specimens were collected. Other rats (*n* = 20) were used to observe the survival rate after tracheal inoculation with *P. aeruginosa*. A solution of 3 % sodium pentobarbital (30 mg/kg) was used to induce intraperitoneal anesthesia, and then a tracheotomy was performed to inoculate rats with bacteria (0.2 ml ATCC27835, 6 × 10^8^ cfu/ml) to induce *P. aeruginosa* lung infection.

### Sample Collection

Rats were euthanized at 0 h (before inoculation) or at 2, 6, 9, 12 and 24 h after inoculation and specimens were taken. At the corresponding time points, we used 3 % sodium pentobarbital (30 mg/kg) for intraperitoneal anesthesia, and performed thoracotomy under sterile conditions to obtain heart blood and left lung bronchoalveolar lavage fluid. Then we measured whole blood leukocyte and alveolar lavage fluid leukocyte counts. Part of the right lung lower lobe was used for bacteriology, to determine the colony counts. The middle part right lung heart leaf was placed in 10 % formalin, fixed and used for histology. The remaining lung tissue was frozen and kept at −80 °C for chemokine gene expression by real-time PCR.

### Etiological Diagnosis

Approximately 5 × 5 × 5 mm lung tissue was placed into a grinder to obtain lung tissue homogenates, which were then placed in blood agar at 37 °C for 24 h. The successful establishment of the pneumonia model depends on the bacteriological index (lung homogenates colony counts) and histopathological examination. Modeling standards used previous definitions [7] as follows: the acute phase, (1) lung tissue bacterial content > 10^5^ cfu/g, (2) histological examination, pathological changes include: pulmonary edema, fiber protein exudation, neutrophil infiltration; chronic phase: (1) lung tissue bacterial content > 10^3^ cfu/g, (2) histological examination, pathological changes in the consolidation, including lung tissue, fibrosis, lymphocytic infiltration. Model standards were also defined according to Song and Model ZJ as previously described [8, 9]. The inflammatory cell infiltration from observed lesions mainly contained polymorphonuclear (PMN) leukocytes responding to acute inflammation, and mononuclear cells accompanied by lymphocytes, red blood cells and granulomatous responses induced by chronic inflammation.

### Whole Blood Leukocyte Count

Whole blood leukocyte counts were performed using the CELL DYN3700 automatic blood cell analyzer detection mode of mammals [8].

### Pulmonary Histopathological Examination

The right lung heart leaf was placed in 10 % formalin to be fixed, then embedded in paraffin and sectioned 6 μm for hematoxylin and eosin (HE) staining. The slices were observed by microscope, and PMN and monocytes/macrophages were counted by optical microscope count under 400-fold magnification (field of view equivalent to 20,000 μm^2^). Ten areas from each section were assessed double-blind. Results were expressed as the number of cells per unit area.

### Expression of CINC and MCP-1 by RT-PCR

#### Extraction of Total RNA

RNA was obtained by Trizol extraction, and agarose gel electrophoresis was used for quality testing.

#### cDNA Reverse Transcriptase

Diluted RNA samples were used for reverse transcription. The reaction system included: 5× reverse transcriptase buffer 4 μl, oligo(dT) 0.5 μl; dNTPs, 0.5 μl; reverse transcriptase MMLV 1 μl; DEPC-treated water 10 μl; and RNA Template 4 μl. The total volume was 20 μl. The reaction conditions were as follows: 37 °C for 1 h, then 95 °C for 5 min to inactivate MMLV.

#### Real-Time PCR

We used real-time PCR to detect gene transcription of CINC and MCP-1 mRNA from different groups. Primer sequences were as follows: GAPDH: (5′ primer): 5′-CTCTACCCACGGCAAGTTCAA-3′, (3′ primer): 5′-GGATGACCTTGCCCACAGC-3′, amplified product length 515 bp; CINC sequence: (5′ primer): 5′-AACAGAGCACCATGGTCT-3′, (3′ primer): 5′-GACGCCATCGGTGCAATCTA-3′, amplification product length 337 bp; MCP-1 sequence: (5′ primer): 5′-CTCTTCCTCCACCACTATGC-3′, (3′ primer): 5′-CTCTGTCATACTGGTCACTTC-3′, amplification product length 457 bp. Then the prepared cDNA was amplified by PCR as follows: SYBRGreen Mix 32.5 μl; upstream primer F 0.5 μl; downstream primer R 0.5 μl; ddH2O 14.5 μl; cDNA Template 2 μl, for a total volume of 50 μl. The reaction mix was placed in an ABI-7500 (ABI, USA) PCR thermal cycler for amplification. The reaction cycle was as follows: (1) denaturation of the initial template: 30 s at 95 °C, one cycle; (2) denaturation of the Template 5 s at 95 °C, annealing for 31 s at 60 °C 40 cycles; (3) melting: 15 s at 95 °C, then 1 min at 60 °C, 15 s at 95 °C (collected fluorescence between 60 °C and 95 °C), then 15 s at 40 °C. The standard curve was obtained by logarithmic fitting the CT value. The following equation was used to calculate the amplification efficiency: *E* = 10(−1/slope). The housekeeper gene, GAPDH, was used as an internal control. The relative content of the sample gene = 2 − ∆CT × 100 (∆CT = test gene CT − housekeeper gene CT).

#### Statistical Analysis

Data are presented as mean ± standard deviation (SD). Differences were considered statistically significant when *P* < 0.05. Differences between groups of rats were determined by non-parametric tests.

## RESULTS

### *P. aeruginosa-*Induced Pulmonary Infection

One hour after inoculation with *P. aeruginosa*, there was no significant effect in either of the two groups, possibly due to the effect of the anesthetic. At 2–4 h later, the young group showed brief breathing, frequent nose scratching and a cough that gradually increased in frequency. All these symptoms leveled off during 6–9 h after inoculation. At 9 h, the young rats become more active and their respiratory rate began to reduce. At 24 h, the young rats performed similarly to preoperative levels. At the time of *P. aeruginosa* inoculation, the aged rat group demonstrated shallow breathing, frequent scratching of the nose, and significant coughing. At 12–16 h, all these symptoms had leveled off but were not restored until 24 h later. The aged rats appeared to move slowly and eat less compared with the young rat group (Tables 1 and 2).

**Table 1:** Comparing the Nose Scratching (Symptom) of Rats Between Two Groups After Inoculation of *P. aeruginosa*

Time point (h)	Young group (number of nose scratching/the total number)	Aged group (number of nose scratching/the total number)
0	(0/10)	(0/10)
1	(5/10)	(5/10)
2	(10/10)	(10/10)
6	(9/10)	(10/10)
9	(7/10)	(8/10)
12	(6/10)	(6/8)
24	(1/10)	(5/7)
48	(0/10)	(0/6)

**Table 2:** Comparing Cough (Symptom) of Rats Between Two Groups After Inoculation of *P. aeruginosa*

Time point (h)	Young group (number of cough/the total number)	Aged group (number of cough/the total number)
0	0/10	0/10
1	3/10	5/10
2	5/10	10/10
6	10/10	10/10
9	6/10	8/10
12	3/10	7/8
24	1/10	5/7
48	0/10	0/6

### Survival

**Fig. 1:**
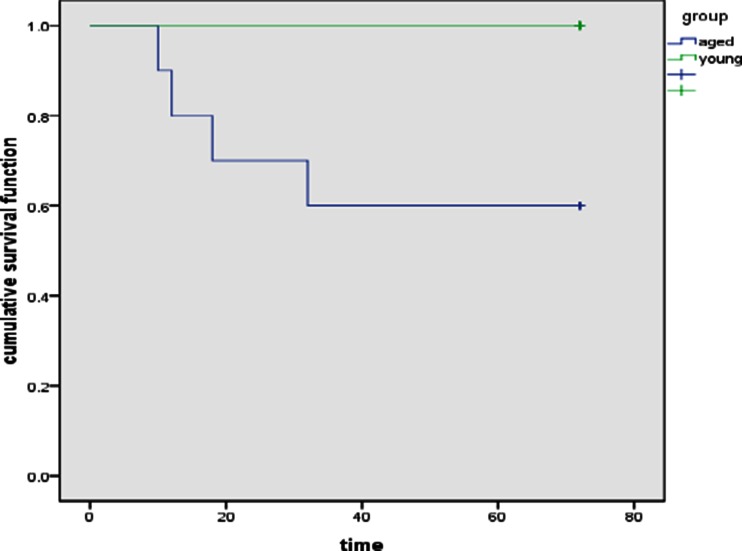
Comparison of cumulative survival rates between two groups.

Rats were observed for 7 days. All rats from the young group survived whereas four rats in the aged group died (40 %), one each at 10, 12, 18 and 32 h after inoculation. We use the Kaplan–Meier to describe the survival (Fig. 1).

### Bacteriological Counts of Lung Tissue

After inoculation with *P. aeruginosa*, the bacterial load in lung tissue showed a downward trend in both groups. At 24 h, it reached minimum levels. However, there was a slight increase at 6 h in the young group. At 2, 6, 9, 12 and 24 h after *P. aeruginosa* inoculation, the bacterial load in lung tissue from the older group was significantly higher than the young group (*P* < 0.001–0.041) (Table 3).

**Table 3:** Dynamic Changes of Bacterial Load in Lung Tissue Before and After Inoculation of *P. aeruginosa* in Two Groups of Rats

Time point (h)	Young group (× 10^4^ cfu/ml mean ± SD)	Aged group(× 10^4^ cfu/ml mean ± SD)	*t* ^a^	*P* value
2	9.75 ± 2.68	23.95 ± 3.93	−5.970	0.001
6	10.95 ± 2.12	19.53 ± 4.90	−3.337	0.034
9	8.56 ± 0.63	13.10 ± 1.69	−5.043	0.002
12	7.31 ± 1.11	11.96 ± 2.15	−3.870	0.008
24	2.13 ± 1.72	5.82 ± 2.27	−2.593	0.041

Data presented as mean ± SD

^a^Non-parametric test

### Peripheral Blood White Blood Cell Counts

Before inoculation with *P. aeruginosa*, the total number of white blood cells in the peripheral blood of the old group was significantly lower than in the young group (*P* < 0.015). After inoculation with P*. aeruginosa*, the total number of white blood cells in the peripheral blood of both groups increased and then decreased. The young group showed a peak at 9 h, which then declined and increased again at 24 h. The old group showed a peak at 12 h (the peak was lower than in the young group), which then declined slowly. At 24 h, it was still approximately four times higher than the normal level. After inoculation with *P. aeruginosa*, the total number of white blood cells in the peripheral blood of the aged group was significantly higher than in the young group (*P* < 0.002–0.024) (Table 4).

**Table 4:** Dynamic Changes of White Blood Cells of Peripheral Blood Before and After Inoculation of *P. aeruginosa* in Two Groups of Rats

Time point (h)	Young group (× 10^9^/l mean ± SD)	Aged group (×10^9^/l mean ± SD)	*t* ^a^	*P* value
0	3.63 ± 0.38	2.83 ± 0.29	−3.337	0.015
2	6.63 ± 1.28	3.43 ± 0.15	−3.593	0.037
6	9.92 ± 2.39	4.65 ± 2.56	−3.010	0.024
9	13.33 ± 2.32	6.48 ± 1.46	−5.012	0.002
12	5.70 ± 1.21	9.20 ± 1.36	−3.846	0.009
24	9.73 ± 2.36	3.67 ± 1.32	−4.663	0.003

Data presented as mean ± SD

^a^Non-parametric test

### Pulmonary Histopathology

Before inoculation with *P. aeruginosa*, lung histology was normal. After inoculation, the lung tissue from the two groups (the upper lobe and the lower lobe) showed varying degrees of edema, hemorrhage and fibrosis by gross examination. The young group showed significant changes between 6 and 12 h that disappeared by 24 h. The aged group showed significant changes between 9 and 24 h. At 24 h, these changes were only slightly reduced compared with at the peak. By endoscopy, lesions in tissues of the young group were limited with inflammatory responses, relatively minor structural damage in lung tissues, and with a peak inflammatory response at 6–12 h. In addition, there were large numbers of PMN and AM (alveolarmacrophage) aggregated in the lesions. In contrast, between 6 and 12 h, lesions of the aged group lungs were diffuse with slight inflammatory response, although edema and hemorrhage were more common.

**Fig. 2:**
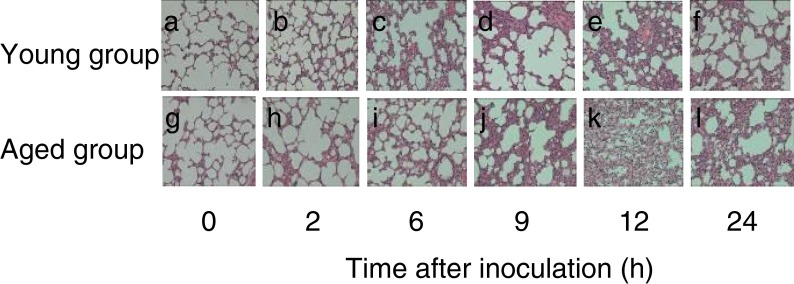
Histology of lung tissue at the indicated time points after inoculation of *P. aeruginosa* in the young and aged group of rats. Magnification, ×400.

Measurement of leukocyte counts in lung tissues demonstrated that before inoculation, there were few aggregated PMN or monocytes/macrophages in the lungs of both groups, and although there were less in the aged group, this was not statistically significant (*P* > 0.05). After *P. aeruginosa* inoculation, the aggregation of PMN and monocytes/macrophages increased. The speed and magnitude of PMN in the young group was higher than in the aged group. The young group reached a peak at 9 h whereas the aged group reached a peak at 12 h, then decreased gradually. For monocytes/macrophages, the young group reached a peak at 12 h, and the aged group reached a peak at 24 h. At each time point, the number of PMN in the lungs of the aged group were significantly lower than the young group (*P* < 0.001–0.010) (Table 5) and the number of monocytes/macrophages in the aged group were also lower than the young group. Differences at 2, 9 and 12 h were statistically significant (*P* < 0.001–0.040) (Table 6, Fig. 2).

**Table 5:** Dynamic Changes of PMN Infiltration into the Lung Tissue Before and After Inoculation of *P. aeruginosa* in Two Groups of Rats

Time point (h)	Young group (20,000 μm^2^ mean ± SD)	Aged group (20,000 μm^2^ mean ± SD)	*t* ^a^	*P* value
0	6.75 ± 0.96	4.75 ± 0.50	0.168	0.010
2	18.50 ± 3.70	9.75 ± 2.50	3.921	0.0087
6	28.25 ± 2.99	18.50 ± 3.87	3.987	0.007
9	55.00 ± 5.77	27.75 ± 3.77	7.901	<0.001
12	53.75 ± 4.79	39.50 ± 4.43	4.367	0.005
24	32.75 ± 3.30	24.50 ± 1.73	4.423	0.004

Data presented as mean ± SD

^a^Non-parametric test

**Table 6:** Dynamic Changes of Monocyte/Macrophages Infiltration into the Lung Tissue Before and After Inoculation of *P. aeruginosa* in Two Groups of Rats

Time point (h)	Young group (20,000 μm^2^ mean ± SD)	Aged group (20,000 μm^2^ mean ± SD)	*t* ^a^	*P* value
0	5.75 ± 1.50	4.50 ± 1.29	−1.263	0.250
2	8.00 ± 0.82	6.75 ± 0.50	2.611	0.040
6	10.50 ± 1.91	8.25 ± 1.26	1.964	0.097
9	14.25 ± 0.96	10.00 ± 0.82	6.755	0.001
12	20.75 ± 1.71	13.25 ± 1.71	6.221	0.001
24	16.75 ± 0.96	15.25 ± 1.71	1.532	0.176

Data presented as mean ± SD

^a^Non-parametric test

### RT-PCR

Before *P. aeruginosa* inoculation, low levels of CINC and MCP-1 mRNA were observed in rat lung tissue from the two groups. However, the transcription levels of MCP-1 mRNA, but not CINC mRNA, were significantly higher in the aged group compared with the young group. With increasing time after inoculation, CINC and MCP-1 mRNA expression increased and was higher in the young group compared with the aged group. CINC mRNA levels reached a peak at 9 h in the young group, and at 12 h in the aged group, and then decreased. At 2, 6 and 9 h after inoculation with *P. aeruginosa*, CINC mRNA expression in the lungs of the aged group were significantly lower than in the young group (*P* < 0.002–0.026). MCP-1 mRNA levels in the young group reached a peak at 9 h and then declined, whereas in the aged group MCP-1 mRNA levels were downregulated at 2, 6 and 9 h after inoculation. The transcription levels of MCP-1 mRNA in the aged group were significantly lower than in the young group (*P* < 0.001–0.014) (Tables 7 and 8).

**Table 7:** Dynamic Changes of Lung Tissue CINC mRNA Expression Before and After Inoculation of *P. aeruginosa* in Two Groups of Rats

Time point (h)	Young group (CINC/GAPDH, mean ± SD)	Aged group (CINC/GAPDH, mean ± SD)	*t* ^a^	*P* value
0	0.52 ± 0.41	0.13 ± 0.05	1.194	0.104
2	1.25 ± 0.34	0.26 ± 0.14	5.393	0.002
6	2.52 ± 0.55	0.45 ± 0.25	6.831	0.002
9	2.05 ± 0.58	0.84 ± 0.58	2.939	0.026
12	1.88 ± 0.16	1.55 ± 0.76	0.720	0.511
24	0.94 ± 0.12	1.01 ± 1.14	−0.109	0.920

Data presented as mean ± SD

^a^Non-parametric test

**Table 8:** Dynamic Changes of Lung Tissue MCP-1 mRNA Expression Before and After Inoculation of *P. aeruginosa* in Two Groups of Rats

Time point (h)	Young group (MCP-1/GAPDH, mean ± SD)	Aged group (MCP-1/GAPDH, mean ± SD)	*t* ^a^	*P* value
0	0.56 ± 0.18	0.28 ± 0.10	2.705	0.035
2	0.84 ± 0.11	0.59 ± 0.05	3.722	0.014
6	1.28 ± 0.09	0.83 ± 0.06	7.308	0.001
9	2.22 ± 0.32	1.26 ± 0.20	4.377	0.012
12	1.74 ± 0.13	1.87 ± 0.56	−0.409	0.720
24	1.42 ± 0.02	2.28 ± 1.12	−1.326	0.316

Data presented as mean ± SD

^a^Non-parametric test

## DISCUSSION

Chemokines are a class of small, secreted proteins that attract white blood cells [9–14]. Many studies have shown that chemokines and their receptors play an important role in pulmonary infectious diseases [15–20]. Many recent studies have analyzed the expression of chemotactic factors in the lungs during infection, but there have been few studies performed in aged subjects. Gomez *et al*. [21] inoculated lipopolysaccharide (from *P. aeruginosa*) into the lungs of BALB/c mice and observed that the transcription levels of MIP-2 and KC in elderly mice were markedly higher than in young mice. In addition, PMN infiltration was greater in the elderly group. This indicated that severe systemic inflammatory responses and exacerbated lung inflammation occurred in elderly subjects and might be a mechanism for the high mortality of elderly rats. Another study investigated the difference of immune functions in young and old mice by inoculation of sub-lethal doses of influenza virus into the lungs of mice, and found that the older group had a higher mortality, slower recovery, increased weight loss, and a delay in infiltrating pulmonary local neutrophils and dendritic cells, corresponding to reduced lung chemokine expression [22].

A previous study indicated that *P. aeruginosa* is the most common hospital-acquired pneumonia pathogen, especially in intensive care units, and causes pneumonia in patients with nosocomial pathogens [23, 24], especially in the elderly, and those that are immunocompromised or with underlying diseases. *P. aeruginosa* is a representative pathogen for aging pneumonia. In addition, PMN and monocytes/giant macrophages play an important role in *P. aeruginosa-*induced lung infection *in vitro* and *in vivo*. MCP-1 also has a specific role in monocytes/macrophages recruitment, so we chose to monitor the dynamic changes of these two cell types and the corresponding changes in lung chemokines during infection.

In 2001, a neutropenia rat model of *P. aeruginosa* pneumonia was developed [5]. We followed this modeling approach and determined that tracheal inoculation could establish a *P. aeruginosa-*induced pneumonia model in rats. Relative to the young rat group, elderly rats had a reduced bacterial clearance capacity. The elderly rats demonstrated more severe symptoms with higher mortality, suggesting the aged group had reduced immune defense. In addition, lung edema and hemorrhage were more common in the elderly rats, but with less severe inflammatory responses. Before and after *P. aeruginosa* infection, numbers of PMNs and monocytes/macrophages in the lung of aged rats were lower than in young rats. After *P. aeruginosa* infection in aged rats, the infiltration of inflammatory cells, in particular PMNs, was delayed, suggesting local cellular defense responses were reduced. This was consistent with a previous study [25] that demonstrated the most vulnerable patients to *P. aeruginosa* in hospital were those whose PMNs were reduced or whom were under machine ventilation. Their fatality rates could exceed 30 %. Differences in pathology between our study and that of Gomez might be related to *P. aeruginosa* dose, animal selection, research methodology and different time points examined. Our study proposed to analyze acute inflammatory reactions induced by *P. aeruginosa*, and the selection of time points up to 24 h were based on a previous study [5]. At different time points, the infiltration mechanisms of PMNs and mononuclear/macrophage cells may be different. PMN and monocyte/macrophage infiltration in the lungs of rats was time-dependent. The transcription levels of CINC and MCP-1 genes (rCINC young group = 0.622, *P* = 0.188; rCINC aged group = 0.970, *P* = 0.001; rMCP-1 young group = 0.757, *P* = 0.081; rMCP-1 aged group = 0.997, *P* < 0.001) were consistent with a previous study [23] that demonstrated CINC and MCP-1 gene expression was reduced in the aged rats group. Thus, aging may cause reduced CINC and MCP-1 gene expression levels, which would weaken the speed and extent of infiltration of PMNs and monocytes/macrophages into lung tissues and affect their defense functions. The poor prognosis and mechanisms of clinical aging pneumonia require further study.

The current study showed some differences compared with a study on the impact of aging on MCP-1 expression by Lin et al. [26], who used 27-month-old female Wistar rats and fibrin to induce acute lung injury. Their study demonstrated that aging contributed to fibrin deposition and enhanced the expression of MCP-1 in lung tissue, which may explain why elderly individuals are more susceptible to inflammatory stimuli. Differences in study findings are probably due to experimental animal selection and the method of establishing the disease model.

*P. aeruginosa* pneumonia is a common cause of death in the elderly. Effective host defense responses remove pathogenic microorganisms in the host lung tissue. This requires the infiltration of PMNs and monocytes/macrophages to the lung [26, 27] attracted by the presence of secreted chemokines.

This research has several limitations. Firstly, we did not conduct a dose gradient experiment. Instead, we just used the common dose used at home and abroad to induce the model of pulmonary infection in young rats to investigate the feasibility in inducing aged rats model. Therefore, the deficiency in this experiment is not to compare between different modeling dose in order to provide a better choice.

Secondly, considering the important role of PMN and macrophage in *P. aeruginosa* pneumonia, we chose to monitor the dynamic changes of these two kinds of cells and the corresponding chemokines (CINC, MCP-1) in pulmonary. However, huge inflammatory cells and chemokines participate in clinical pulmonary inflammation and the concerned regulation mechanism is rather complex. Thus, the advanced mechanism should be studied further.

Thirdly, we studied the expression levels of CINCmRNA and MCP-1mRNA, but the analysis of its protein level was not carried out in our study as to whether the reduction of gene transcription levels in early infection affects protein expression and function. This can be studied in our further research.

Fourthly, the experiments only simulated and investigated the mechanism of acute *P. aeruginosa* pneumonia. So whether we will have the same conclusion concerning chronic *P. aeruginosa* pneumonia still needs to be studied.

In summary, this study demonstrated that aging can reduce the expression of CINC and MCP-1 mRNA in lung tissues, and thus can reduce the infiltration of PMNs and monocytes/macrophages induced by CINC and MCP-1, which may lead to increased risk of pneumonia in the elderly. The molecular and regulatory mechanisms involved require further study.
